# Duetting Patterns of Titi Monkeys (Primates, Pitheciidae: Callicebinae) and Relationships with Phylogeny

**DOI:** 10.3390/ani8100178

**Published:** 2018-10-13

**Authors:** Patrice Adret, Kimberly A. Dingess, Christini B. Caselli, Jan Vermeer, Jesus Martínez, Jossy C. Luna Amancio, Silvy M. van Kuijk, Lucero M. Hernani Lineros, Robert B. Wallace, Eduardo Fernandez-Duque, Anthony Di Fiore

**Affiliations:** 1 Museo de Historia Natural Noel Kempff Mercado, Santa Cruz de la Sierra 2489, Bolivia; 2 Department of Biological Sciences, Marshall University, Huntington, WV 25755, USA; 3 Departamento de Biologia, Universidade Federal Rural de Pernambuco, Recife 52171-900, PE, Brazil; 4 Proyecto Mono Tocón, Moyobamba 22001, Peru; 5 Wildlife Conservation Society, La Paz 3-35181, Bolivia; 6 Department of Anthropology, University of Texas, Austin, TX 78712, USA; 7 Carrera de Biología, Universidad Mayor de San Andrés, La Paz 6042, Bolivia; 8 Department of Anthropology and School of Forestry & Environmental Studies, Yale University, New Haven, CT 06520, USA

**Keywords:** *Plecturocebus*, *Cheracebus*, *Callicebus*, vocal communication, taxonomy, conservation

## Abstract

**Simple Summary:**

Titi monkeys—a diversified group of pair-bonded, territorial neotropical primates exhibiting biparental care—produce elaborate, powerful vocal duets used for long-range communication. While the callicebine taxonomy has been centered mainly on the biogeography, morphology, anatomy, and genetics of titi populations, vocal attributes have received little attention as potentially informative markers of phylogenetic relationships. We conducted acoustic analysis of callicebine loud calls recorded from ten species of titis at sites in Bolivia, Peru, and Ecuador and found four distinct patterns of duetting that only partially match three major clades identified in recent molecular genetic studies. In particular, we found that the loud calls of the San Martin titi monkey, *P. oenanthe*, and the Urubamba brown titi, *P. urubambensis*, strikingly differ from putative relatives within the donacophilus lineage. Our findings highlight interplay between genes and environment on the expression of vocal behavior and suggest that closer interaction between taxonomists, ethologists, and molecular biologists should be rewarding in resolving the callicebine phylogeny. Such concerted efforts, in turn, will most likely generate valuable recommendations for the conservation of some endangered populations of titi monkeys, such as the vocally distinctive San Martin titi.

**Abstract:**

Long-range vocal communication in socially monogamous titi monkeys is mediated by the production of loud, advertising calls in the form of solos, duets, and choruses. We conducted a power spectral analysis of duets and choruses (simply “duets” hereafter) followed by linear discriminant analysis using three acoustic parameters—dominant frequency of the combined signal, duet sequence duration, and pant call rate—comparing the coordinated vocalizations recorded from 36 family groups at 18 sites in Bolivia, Peru and Ecuador. Our analysis identified four distinct duetting patterns: (1) a donacophilus pattern, *sensu stricto*, characteristic of *P. donacophilus*, *P. pallescens*, *P. olallae*, and *P. modestus*; (2) a moloch pattern comprising *P. discolor*, *P. toppini*, *P. aureipalatii*, and *P. urubambensis*; (3) a torquatus pattern exemplified by the duet of *Cheracebus lucifer*; and (4) the distinctive duet of *P. oenanthe*, a putative member of the donacophilus group, which is characterized by a mix of broadband and narrowband syllables, many of which are unique to this species. We also document a sex-related difference in the bellow-pant phrase combination among the three taxa sampled from the moloch lineage. Our data reveal a presumptive taxonomic incoherence illustrated by the distinctive loud calls of both *P. urubambensis* and *P. oenanthe* within the donacophilus lineage, *sensu largo*. The results are discussed in light of recent reassessments of the callicebine phylogeny, based on a suite of genetic studies, and the potential contribution of environmental influences, including habitat acoustics and social learning. A better knowledge of callicebine loud calls may also impact the conservation of critically endangered populations, such as the vocally distinctive Peruvian endemic, the San Martin titi, *P. oenanthe*.

## 1. Introduction

Animal communication is the process by which a listener acquires information from a sender or changes its behavior to the benefit of the signaler [1,2]. All primates—strepsirhines, tarsiers, Old and New World monkeys, apes, and humans—communicate by means of visual, auditory, chemical, and tactile signals [3]. Signaling is key to survival and fitness, as, for instance, in the context of sexual reproduction, foraging, intergroup spacing, and predator avoidance [4]. Often, more than one communication channel is used, perhaps to increase detectability in a challenging environment or to convey additional information [5,6,7]. However, the predominance of one modality over another likely co-evolved with a species’ circadian activity, its diet and group size, and the habitat structure in which that species lives [8,9]. For diurnal, arboreal primates exploiting dense tropical forests, selection has favored long-range vocal communication over visual signaling as communicating in the visual domain is less effective owing to dense vegetation [10,11].

Many primates produce loud, early morning calls, just before or after dawn, a time when atmospheric conditions are optimal for sound transmission [11,12,13]. These long and intricate vocal displays, often produced jointly by two or more members of a group, and sometimes echoed by similar calls from neighboring groups, represent a special class of acoustic signals referred to as “duets” and “choruses”, respectively [14,15,16,17,18,19,20], and they are presumed to be related to resource defense [3,4,5].

Vocal duets and choruses are among the most intricate signals of acoustic communication in non-human animals [18,21,22]. These joint vocal emissions differ from randomly overlapping sound signals, such as mobbing calls to predators, in that they are both coordinated and stereotyped vocalizations, particularly in insects and birds [7,23,24,25,26]. A duet is created when one animal (the “follower”) joins another who initiated calling (the “leader”) to produce a dual signal whose form and structure can be so precise that a human listener may perceive it as the output of a single individual [26,27,28]. Avian duetting is taxonomically widespread [29,30], and, to date, birds provide the best source of knowledge with respect to duet function, evolution, causation, and ontogeny [28,29,30,31,32,33]. Since Wickler’s influential work [34] proposing a simple mechanism that links pair-specific duetting to pair bonding, various hypotheses have been formulated (and tested on numerous species) in terms of cooperation and conflict between the two partners to explain the functions of avian duetting [35]. 

Much less is known about duetting in mammals in which the behavior is found mainly, although not exclusively [36], in primates with a socially monogamous and territorial lifestyle such as the Mentawai langur (*Presbytis potenziani*), a lemur, the Indri (*Indri indri*), Sulawesi tarsiers (*Tarsius* spp.), Southeast Asian gibbons (*Hylobates* spp., *Nomascus* spp., *Symphalangus syndactylus*, and *Bunopithecus hoolock*), and neotropical titi monkeys (*Plecturocebus* spp., *Cheracebus* spp., and *Callicebus* spp.) [21,37,38,39,40,41,42,43,44,45,46,47,48]. The temporal pattern of these combined signals varies from antiphonal, non-overlapping duetting, as in gibbons [39], to simultaneous, overlapping duetting, as in titi monkeys [44]. Although not all family living, pair-bonded primates exhibit duetting behavior, most of those that do are sexually monomorphic in body size, with little or no sex difference in vocal output [49]. The functional significance of primate duetting has been hypothesized to signal the mated status of one or both pairmates (the “mate guarding” and “pair bond maintenance” hypotheses) and/or to advertising occupancy and willingness to defend an area in space that contains food or other resources (the “joint territory defense” hypothesis) [50,51].

**Figure 1:**
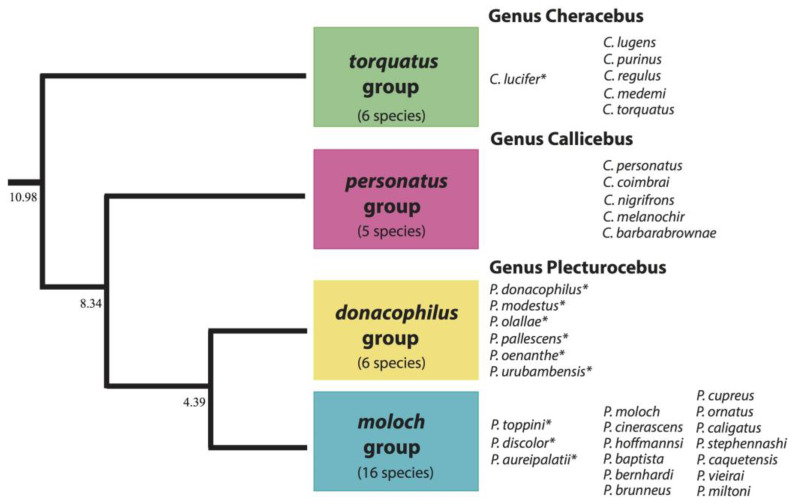
Phylogenetic reconstruction based on mitochondrial, nuclear, and combined datasets of high quality DNA showing the divergent times (mean age in Ma) of the four clades of titi monkeys. Shown at right is the current taxonomy at the genus level and with 33 species. Asterisks denote the ten species of titis investigated in the present study. Adapted from Byrne et al. [62].

Titi monkeys (Pitheciidae; subfamily Callicebinae) are a monophyletic clade of pair-bonded species exhibiting biparental care [52,53,54,55] and in which the taxonomy remains contentious [56,57,58,59,60,61,62,63,64,65,66], with several taxa being re-evaluated and/or newly described within last two decades [59,67,68,69,70,71,72]. Until recently, our understanding of the taxonomy and phylogeny of titi monkeys was based mainly on biogeographical, morphological, and anatomical criteria complemented by chromosome analysis [73,74,75,76]. However, recent molecular studies involving multiple genetic markers [62,63,64,65,66] have prompted a reassessment of phylogenetic and biogeographic relationships among the various titi taxa, leading Byrne et al. [62] to split the former genus *Callicebus* into three genera, including *Plecturocebus* (with 22 species) and *Cheracebus* (with six species), which together are distributed in the Amazon and Chaco ecoregions, and *Callicebus* (with five species), which is endemic to eastern Brazil. These authors, in addition, provide genetic evidence for the existence of four major lineages within the subfamily: the moloch and donacophilus groups (both within the genus *Plecturocebus*), the personatus group (genus *Callicebus*), and the torquatus group (genus *Cheracebus*), which is the most basal lineage within the callicebine radiation (Figure 1). While multilocus genetic data are crucial for elucidating phylogenetic relationships with higher resolution than afforded by morphological, anatomical, and cytogenetic data, it is important to consider the extent to which behavioral traits such as long-range vocalizations (“loud calls”) can also be potential indicators of species-specificity and phylogenetic affinity, as has been reported for songbirds, tarsiers, tamarins, and gibbons [30,43,46,77,78,79].

To date, relatively little is known about duetting in titi monkeys. Only seven of the 33 callicebine taxa that are currently recognized [62] have been the focus of research on either the structure and organization of loud calls (*Plecturocebus discolor* [37], *P. ornatus* [38], and *P. cupreus* [37,44] in the moloch group; *Callicebus nigrifrons* [48] in the personatus group; and *P. oenanthe* [80] in the donacophilus group) or on their functional significance (*P. ornatus* [81,82], *Cheracebus lucifer* [83,84], *P. brunneus* [50], now considered as *P. toppini* [72], and *C. nigrifrons* [51]). These bioacoustics reports, however, remain incomplete owing to the complexity of the joint vocal performance in titis. For instance, in contrast to the tonal utterances and vocal alternations that are readily apparent in gibbon duets [43,85], the joint acoustic displays of titi monkeys are characterized by considerable overlap of broadband calls between the two partners (and sometimes additional individuals, such as a putative offspring of the pair), which is problematic for detailed sound analysis. As a result, previous studies tended to focus on those portions of duet calls showing little or no overlapping sounds or used a subset of duet recordings for which the calls of one pair member were louder and clearly distinguishable on the spectrogram [38,44,49]. Overall, the lack of suitable techniques for separating individual voices that overlap extensively during joint vocal performances has hampered progress in describing the structural and organizational features of titi vocalizations [86].

Here, we describe the acoustic structure of titi monkeys loud calls—emitted mostly as duets—in the donacophilus lineage (genus *Plecturocebus*), for which little information is available [80]. For comparative purposes, we also describe the homologous loud calls of the Red titi monkey (*Plecturocebus discolor*), the Toppin’s titi monkey (*P. toppini*), and the Golden Palace titi monkey (*P. aureipalatii*) from the moloch lineage as well as the duet of the Yellow-handed titi monkey (*Cheracebus lucifer*) from the torquatus lineage (Figure 1). Given the existence of distinct patterns of duetting among these lineages, an attempt is made to relate such vocal diversity to the current callicebine taxonomy, which is based on genome-wide marker data and to the potential influence of environmental variables, including social learning. We close with a brief discussion on the implications of our results for the conservation management of titi monkey populations.

As we show below, we uncovered substantial differences in both the spectral features and the temporal organization of long sequences emitted mostly as duets among those three lineages of titi monkeys. In particular, we found that the duetting patterns of the San Martin titi monkey, *P. oenanthe*, and the Urubamba brown titi, *P. urubambensis*, differ strikingly from other putative members within the donacophilus group. We also report subtle but consistent sex-related differences in call structure in the moloch lineage. We feel these observations are of major interest, which warrants their documentation to stimulate further research on the vocal phylogeny of this complex and highly diversified set of genera [87].

## 2. Materials and Methods

### 2.1. Video and Sound Recordings

**Figure 2:**
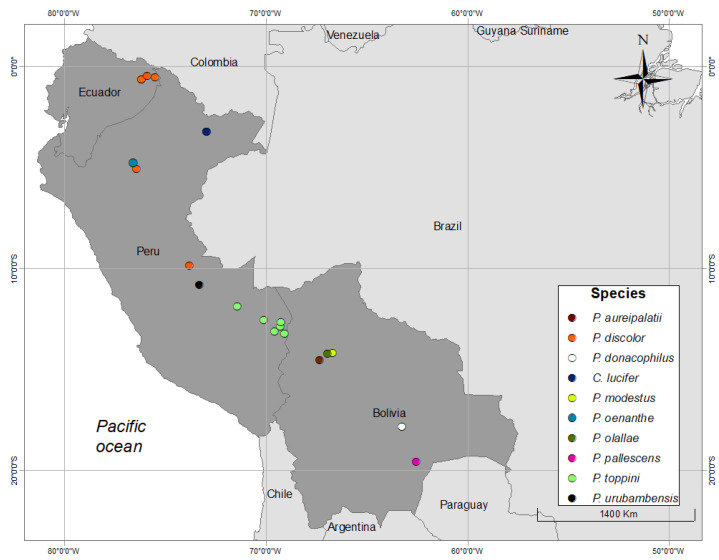
Map showing the 18 recording locations of *Plecturocebus* and *Cheracebus* loud calls that were analyzed in this study.

For this study, we used recordings of wild titis collected by the authors and collaborators in the field between 2008 and 2017. We supplemented these with all suitable archival recordings of titis curated in the Macaulay Library Sound Archive at Cornell University. The 18 recording sites that provided suitable audio material for the present study were distributed in Bolivia (*n* = 5), Peru (*n* = 10), and Ecuador (*n* = 3) (Figure 2). 

#### 2.1.1. Field Recordings in Bolivia

Bolivia harbors six species of titi monkeys distributed both in the donacophilus (*P. donacophilus*, *P. modestus*, *P. olallae*, and *P. pallescens*) and moloch (*P. aureipalatii* and *P. toppini*) lineages [72,88,89,90,91,92,93,94,95,96].

In 2012, we obtained loud call recordings from the Bolivian gray titi monkey (*P. donacophilus*) in the Ecological Park of Yvaga Guazú (S14°14′30″, W66°58′39″) near Santa Cruz de la Sierra. Five habituated family groups (three to six individuals per group) were filmed and recorded opportunistically between 6:00 h and 12:00 h, with occasional footage in the afternoon. Video and audio recordings from two other habituated groups were obtained in 2017 and 2018.

In 2012 and 2013, we obtained video footage and recordings of the two Bolivian endemics *P. modestus* and *P. olallae* during five weeks of fieldwork. In April 2012, we sampled one habituated family group (four individuals) of the Beni titi monkey, *P. modestus*, at San Miguel cattle ranch (S14°1′2.06″, W66°41′58.23″). In April 2012 and May 2013, we sampled loud calls of two nonhabituated groups of Olalla’s titi monkeys, *P. olallae*, at La Asunta cattle ranch (S14°13′49.17″, W66°56′56.65″), on the northern bank of the Yacuma river.

In September 2014, we obtained video and audio recordings of two nonhabituated family groups of the White-coated titi monkey, *P. pallescens*, encountered in a riparian forest on the western bank of the Parapeti river during a one-week stay at La Brecha (S19°30′22.43″, W62°33′40.35″) among Guarani communities of the Isoso territory.

In all these instances, the titis vocalizations were recorded on Panasonic HDV mini-cassettes using a shotgun microphone (Sennheiser ME-66) mounted on a video camcorder (Canon XL-H1). The sampling rate was 48 kHz with a 16-bit sound accuracy. The video material was uploaded in the software Final Cut Pro (v.10, Apple Inc., Cupertino, CA, USA) and relevant sections of the stereo audio track were exported in the WAV format.

Additionally, in 2014, loud call recordings were obtained from a nonhabituated family group of *P. aureipalatii* (two adults and one juvenile) encountered on the western bank of the Hondo river in the Madidi National Park (S14°37′31.73″, W67°43′24.47″). Vocalizations were recorded at a sampling rate of 96 kHz and with a 24-bit sound resolution, using a unidirectional microphone (Sennheiser ME-67, Sennheiser, Wedemark, Germany) connected to a digital tape-recorder (Marantz PMD-671, Marantz, Kawasaki, Japan).

#### 2.1.2. Field Recordings in Ecuador

In 2016, we recorded loud calls of the Red titi monkey (*P. discolor*) at the Tiputini Biodiversity Station in Amazonian Ecuador (S0°38′18″, W76°9′0″). Audio recordings of duets were obtained from four family groups of titis (one habituated and three nonhabituated) with a directional microphone ME-67 connected to a Marantz PMD-660 digital recorder. The sampling rate was 44.1 kHz with a 16-bit resolution. For three of these groups, playback was used to elicit calling. 

#### 2.1.3. Field Recordings in Peru

In 2017, we obtained loud call recordings of the San Martin titi monkey, *P. oenanthe* in a protected area (S5°59′22.14″, W77°02′39.13″). Of the three recordings, two contained vocal duets from a nonhabituated family group (four individuals). The third recording was a duet/chorus recorded from that same group during a territorial dispute with a neighboring group. Vocalizations were captured with a portable digital recorder TASCAM DR-100 mkII and an MKE 600 microphone. The sampling rate was 96 kHz with a 16-bit sound accuracy. 

Additional audio material from *P. discolor* (*n* = 2) and *P. urubambensis* (*n* = 1) was obtained in 2013 from three nonhabituated groups videotaped in northern (S6°32′54”, W76°25′33″) and central Peru (S10°2′50.00″, W74°0′26.00″ and S10°48′50.00″, W73°17′8.00″). The sampling rate was 44.1 kHz, with a 16-bit resolution.

#### 2.1.4. Macaulay Library Sound Archive

Sixteen recordings contributed by nine recordists over a period of 29 years (1980–2009) were downloaded from the pitheciid catalogue of the Macaulay Library (Table 1, Appendix). Titi monkey loud calls were recorded in Ecuador (*n* = 3) and Peru (*n* = 13). The subjects were reported as *Callicebus moloch* (*n* = 11), *C. cupreus* (*n* = 3), and *C. torquatus* (*n* = 2), but these are now considered to belong to different genera and species [57,62,72]. *Cheracebus lucifer* is the only member of the torquatus group for which recordings were available from the Macaulay Library and all came from one location in Peru (Table 1).

Depending on the author, sound was sampled at either 44.1 kHz or 48 kHz with a 16- or 24-bit resolution, respectively. The duration of those recordings ranged from 64 to 318 s, providing a total of 43 min of sound material. For most recordings, information about locality, terrain elevation, estimated group size, group height, and distance from the group was reported, together with a brief description of the habitat and vocal context. Recording equipment included a variety of tape recorders (Nagra IV-B, IV-D, 4.2, Sony TC-D5M, TCM-5000, Marantz PMD-660), microphones (Electro-Voice DL42; Sennheiser ME-80, ME-88, MKH-60, MKH-815; Dan Gibson P-200, P-204), and parabolic reflectors (Saul Mineroff MGA).

### 2.2. Acoustic Terminology

**Figure 3:**
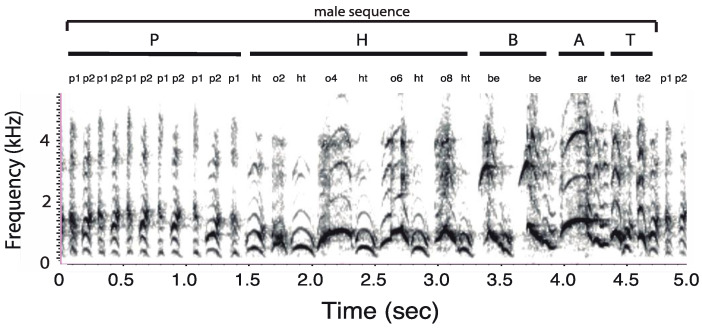
Hierarchical organization of a male *Plecturocebus donacophilus* solo song. The spectrogram shows a sequence composed of five song phrases depicted by horizontal bars. Each phrase consists of consecutive paired syllables, exhibiting some variation in syllable structure (o2, o4, o6, and o8). Individual syllables have been labeled with small letters and numbers. P: pant phrase; H: hoot phrase; B: bellow phrase; A: arch phrase; T: terminal phrase.

For the most part, we adopted the terminology used by previous authors to describe titi calls [37,38,44,48,50,52,80,83,86,97]. However, sounds that did not match others’ descriptions were classified according to our own scheme (Table 2). Here, a “syllable” is a continuous tracing on the spectrogram. A syllable may consist of two or more components referred to as “notes” [98]. For instance, the *Plecturocebus* scream contains three notes, each having distinct spectral characteristics [86]. During the production of loud calls, most syllables are produced in pairs, concomitantly with a rapid pattern of inhalation and exhalation. Loud call emissions consist of runs of similar pairs of syllables assembled into “phrases” that are combined to form a “sequence” [44,48] (Figure 3). A set of song sequences defines a “bout” and thus represents a higher level of song organization. Consecutive bouts are separated from one another by a silent interval (>10 s). A similar pattern of recurring syllables, phrases, sequences, and bouts occurs when two or more individuals join together to produce a coordinated “duet” or a group “chorus” that may last five minutes or more [37,83]. In this case, the moment-to-moment combination of distinct, overlapping sounds between the two pair members (and their offspring, occasionally) generates another level of complexity. A few introductory syllables, consisting of high-pitched syllables and/or moans (Table 2), usually precede the onset of the first duet sequence. Because the introductory part of the duet was missing from two thirds of the recordings, this section was excluded from the analysis.

### 2.3. Acoustic Analysis

We analyzed video and audio recordings of loud calls from ten taxa distributed in three lineages of the callicebine subfamily (Figure 1). The duets of 36 putative family groups were analyzed in this study. To avoid pseudoreplication, we selected only one long bout of duet or chorus per group (Table 3). Raw stereo WAV files were split to mono with the sound software Audacity (v. 2.2.1) and only one channel was used for the acoustic analysis. Audio files were downsampled to 44.1 kHz when necessary and a high-pass filter was applied to eliminate noise below 80 Hz, which did not affect the fundamental frequency (>100 Hz) of the titi monkeys’ voice. To ensure that the loudest peak in each file was the same, all sound files were normalized (SoX, v. 14.4.1, SoundExchange, Washington, DC, USA). The WAV files were then analyzed with the ‘seewave’ package (v. 2.0.5, National Museum of Natural History, Paris, France) [99] in the R environment (The R Foundation for Statistical Computing, v. 3.3.3, R Core Team, Vienna, Austria) [100]. Spectrograms were analyzed in Audacity, using the Hanning filter with a Fast Fourier Transform window size of 2048 points. For display, spectrograms were prepared with the Raven Pro software (v1.5, Cornell Laboratory of Ornithology, Ithaca, NY, USA) using a window size of 1024 points.

Our primary goal was to explore how well the titis’ duets match the current taxonomy. To that effect, we conducted a linear discriminant analysis (see Section 2.4) using three acoustic parameters chosen to maximize the chances of allocating each species’ vocal phenotype to its respective group membership (lineage). Below, we explain the various steps that allowed us to extract the dominant frequency, sequence duration and call rate from our samples. In our quantitative analysis, we consider *P. urubambensis* as part of the moloch lineage, given shared acoustic features with the duets of other species in this clade.

#### 2.3.1. Power Spectrum of the Joint Signal

From each recording, we selected a one-minute subsample of continuous duetting (*n* = 33), avoiding, when possible, noisy sections of the recording (see below). Three recordings (*P. donacophilus*: *n* = 1, *P. discolor*: *n* = 1, and *C. lucifer*: *n* = 1; 8.3%) did not meet the one-minute criterion but were included in the analysis, yielding a total of 36 recordings. To determine the dominant frequency, we generated a power spectrum of the entire signal, using the seewave function ‘meanspec’ (window length: 2048p, overlap = 0). The dominant frequency of the joint signal was defined as the largest peak attributable to that signal and was consistently located within the 80 to 1500 Hz frequency band. For a given taxon, we then computed a power spectrum of the duet recorded from one group only (four taxa) or a mean (± SEMs) power spectrum when more than one group had been recorded (six taxa). Because some of the recordings were contaminated by other sounds—mainly from birds and insects—all recordings were low-pass filtered at 2 kHz using the seewave ‘ffilter’ command. This filtering resulted in a high signal-to-noise ratio, suitable for a cumulative frequency analysis, both within and between species. Using the seewave script ‘diffcumspec’, we then generated cumulative distribution functions from the power spectra of the filtered signal. The spectral difference was measured halfway up the cumulative curves by comparing across taxa the frequency values obtained from each duet.

#### 2.3.2. Dominant Frequency of the Joint Signal

We extracted the dominant frequency of the duet from the one-minute filtered signal (80–2000 Hz), which was the frequency with the highest amplitude value inside that bandwidth. Peak frequency extraction was achieved by using the seewave function ‘fpeaks’, which was set to search for the five largest peaks of the frequency spectrum. The largest of these five peaks was elected as the dominant frequency after visual confirmation of the peak locations along the frequency spectrum. To ensure that our analysis was not affected by signal filtering, we also measured the dominant frequency from a subset of nonfiltered, high-quality calls (*n* = 27). We found no difference in the maximum peak value between the two sets of recordings. Thus, our measures of the dominant frequency from filtered signals are accurate.

#### 2.3.3. Sequence Duration

To quantify duet sequence duration, we proceeded to a manual segmentation of each recorded duet (*n* = 36). We followed the procedure of Müller and Anzenberger [44] but, instead of using bellows as vocal markers of duet sequences, we used pant syllables owing to the absence of bellows in *C. lucifer*. Thus, a “male duet sequence” was defined as the interval separating the first male pant syllable at the onset of a given sequence to the first male pant syllable of the next sequence (Figure 3). Each duet sequence (*n* = 432) was marked on a label track below each spectrogram.

#### 2.3.4. Call Rate

In a subset of male duet sequences (*n* = 387; 89.6%), we measured call rate for pants (*n* = 309), which are produced as paired syllables in all ten species of titis, bellows (*n* = 271), and pumps (*n* = 96). Pumping does not occur in the donacophilus group but we identified analogous phrases that we termed “rhythmic” (Table 2), in which one pair member produces a short sequence of repeated syllables at a high rate (4.1 calls/s), similar to pumping in the moloch group (4.3 calls/s). Thus, rhythmic phrases in the donacophilus group were treated as pump phrases. Call rate within a given phrase was calculated by dividing the number of syllables contained in that phrase (minus one) by the duration from the onset of the first syllable to the onset of the last syllable. Rhythm is an acoustic feature to which the human brain is highly responsive [101], thus, we could identify individual call rate during a duet by carefully listening and annotating the corresponding sounds on the spectrogram. This task was also facilitated, to some extent, by the fact that the calls of titi monkeys appear to be sexually dimorphic [38]. For instance, with some practice, pant syllables are distinguishable by ear between both sexes, with females producing a more nasal sound than males across all taxa. More objectively, in a subset of our video recordings of duets in which both adults were side by side, we could assign sounds produced in synchrony with mouth movements to the sex of each pair member [102].

#### 2.3.5. Sex Difference in Call Structure

To ensure that song syllables had been correctly allocated to a given sex, for seven adult pairs in the moloch lineage (*P. discolor*: *n* = 4; *P. aureipalatii*: *n* = 1; and *P. toppini*: *n* = 2), we extracted 618 exemplars of clearly distinguishable bellows that had been previously assigned to either one of the two pair members. Each newly created WAV file (*n* = 14) contained a collection of loud bellows produced by one pair member and softer pant syllables produced by his/her partner. We then generated power spectra of the fourteen sound files for statistical comparison.

### 2.4. Statistics

The normal distribution of the data and homogeneity of variance was checked with the Shapiro–Wilk test and the Levene test, respectively, prior to using the parametric Student’s *t*-test. With unequal variances, we used the Welch Two Sample *t*-test. When the data did not fit the parametric assumptions, including after logarithmic transformation, we applied the nonparametric Wilcoxon Rank Sum test or the Wilcoxon Signed Rank test for unpaired or paired comparisons, respectively. Where necessary, we applied corrections for multiple testing using the Bonferroni method. Data are shown as mean ± SEMs.

We avoided pseudoreplication by sampling only one duet per group and by performing analyses of acoustic parameters using taxa for which we had recorded from more than one group. To account for multiple measures from a single duet sample, we performed linear mixed effects model analyses using the R package lme4 (v. 1.1-15, R Core Team, Vienna, Austria) in which the song variable was taken as a random effect. In our model, species was the fixed effect and was treated as a categorical factor with six levels.

To determine whether acoustic features of titi monkey loud calls are differentially grouped between the three lineages, we subjected our data to a linear discriminant analysis (LDA). The goal of LDA is to maximize the separation between two or more groups, according to the formula:

(1) $$\frac{{\left(\mu 1-\mu 2\right)}^{2}}{s_{1}^{2}+s_{2}^{2}}$$

where *µ*1 and *µ*2 are the means for each group and the denominator represents the sum of the two variances or scatter. With three predictors, LDA achieves this by creating a new plane (hyperplane) and projects the data on it to (1) maximize the separation between the respective centroids and (2) minimize the scatter around each centroid. Thus, LD1 and LD2 represent the two dimensions for this hyperplane. For our purpose, lineage was the dependent variable and LDA searched for the linear combination of three predictor variables—dominant frequency, sequence duration and call rate—that best separated the three lineages. Because bellows and pumps were not identified in the loud calls of *C. lucifer*, we ran the LDA using the pant syllable, which appears to be homologous across the three lineages.

For this analysis, we proceeded in two steps. First, we ran LDA on our complete dataset (36 duets) with *P. urubambensis* and *P. oenanthe* assigned to the donacophilus lineage, that is, in accordance to the current callicebine taxonomy [62]. We then ran a similar analysis with *P. urubambensis* assigned to the moloch lineage since the species’ duetting pattern conformed to that lineage, as described below. In addition, *P. oenanthe* was removed from the dataset, given its unique vocal phenotype. The discriminant analysis was performed with the R ‘Mass’ package and the resulting biplots were created using the R package ‘ggord’ [103].

The main assumptions of LDA are three-fold: categories must be mutually exclusive, all predictors must be independent and normally distributed, and there should be no outliers. The covariance matrices should also be roughly equal for all groups (lineages). While violation of the normality assumption does not affect substantially the outcome, LDA is especially sensitive to the presence of outliers. The Pearson’s coefficient of correlation was used to test for multicollinearity between the three predictors and the significance of the discriminant analysis was evaluated with a MANOVA, followed by post-hoc comparisons with the Pillai test.

## 3. Results

We begin with a description of the titis’ duetting patterns found in the three lineages, with *P. oenanthe* treated as a distinct vocal phenotype (Section 3.1, Section 3.2, Section 3.3 and Section 3.4). We then move on with analyses of duet power spectra and acoustic parameters leading, in the final step, to a linear discriminant analysis (Section 3.5, Section 3.6 and Section 3.7).

### 3.1. Organization of Duets in the Donacophilus Group

**Figure 4:**
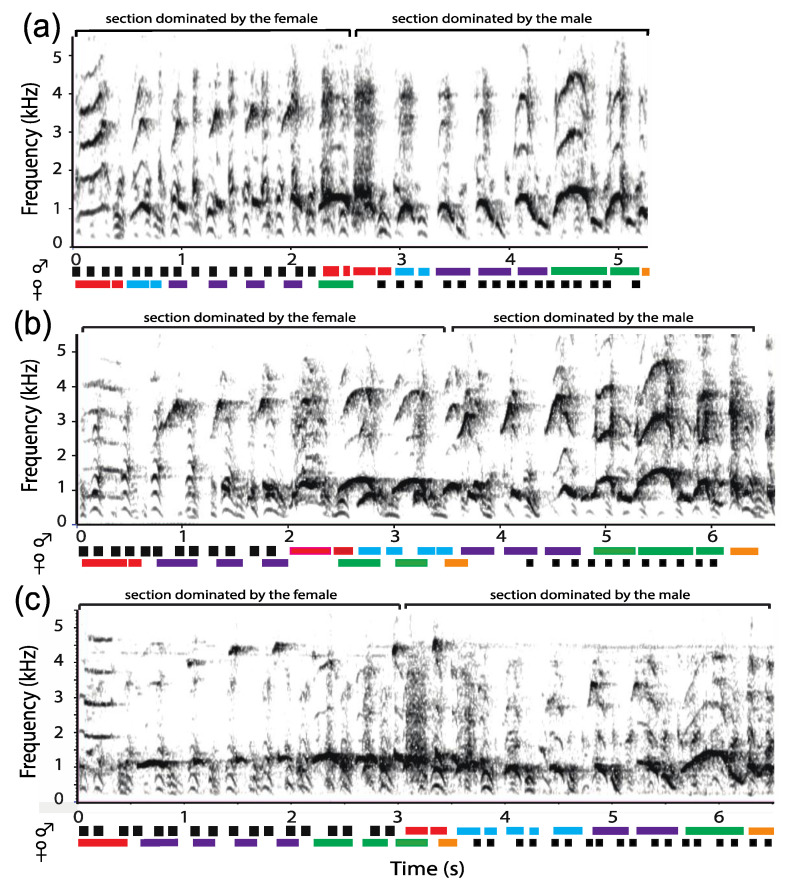
Joint emissions displayed on a short time scale. (**a**) *P. donacophilus*, (**b**) *P. modestus*, and (**c**) *P. olallae*. For each species, the sequence begins at the onset of the male pant phrase. The approximate timing of male and female sounds is represented below each sonogram by colored horizontal bars. Each pair member contributes a sequence of soft, paired syllables (pants and terminal elements; black and orange, respectively) in alternation with a sequence of loud, paired syllables (hoots, bellows, and arches; blue, purple and green, respectively). Correspondingly, in each taxon, we note a female-dominated section followed by a male-dominated section. Loud, noisy syllables and harmonic syllables (shown by red horizontal bars) are vocal markers that characterize the beginning of a male-dominated or female-dominated section. Note the longer duration of a duet sequence in *P. modestus* and *P. olallae*.

**Figure 5:**
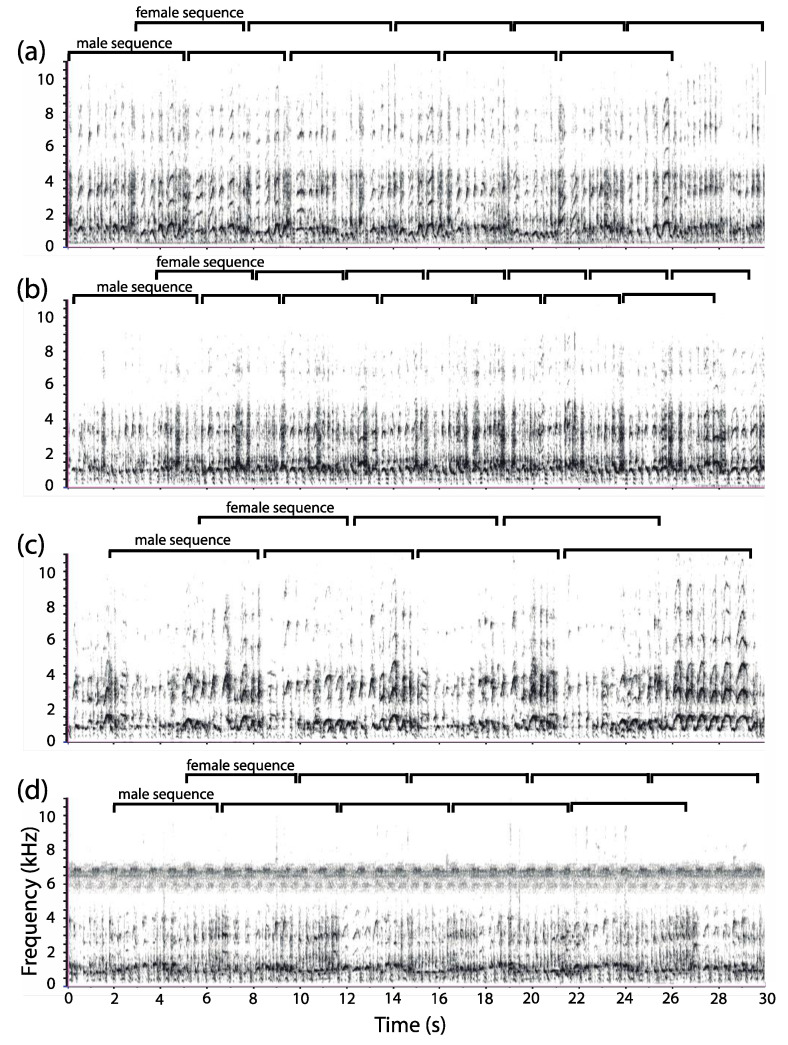
Joint emissions displayed on a larger time scale. (**a**) *P. donacophilus*, (**b**) *P. pallescens*, (**c**) *P. modestus*, and (**d**) *P. olallae*. Time and frequency scales have been adjusted to match the spectrograms representative of the moloch lineage in Figure 6. Putative male and female sequences begin at the onset of the pant phrase produced by each sex, respectively. The spectrogram of *P. olallae* shows noisy insect sounds in the 6 to 7 kHz frequency band.

The coordinated loud calls of *P. donacophilus* (*n* = 7), *P. pallescens* (*n* = 2), *P. olallae* (*n* = 2), and *P. modestus* (*n* = 1) begin with a few high-pitched syllables, occasionally with moans combined. The joint emissions are characterized by sequences of short duration (~5 s) with a high male call rate (*P. donacophilus*: 3.20 ± 0.05 calls/s, *n* = 156; *P. modestus*: 2.99 ± 0.06 calls/s, *n* = 49; *P. olallae*: 3.35 ± 0.08 calls/s, *n* = 39; and *P. pallescens*: 3.38 ± 0.08 calls/s; *n* = 56). Each pair member contributes a sequence of pants, hoots, bellows, arches, and terminal elements that are coordinated to create the duet (Figure 4 and Figure 5). No more than four consecutive bellows and six arches occur during a duet sequence. A female-dominated section precedes the male-dominated section. That is, as the male starts panting, the female produces a loud harmonic syllable (~300 ms) followed by hoots, bellows, and arches. The male then emits a loud, noisy syllable (~200 ms) also followed by hoots, bellows, and arches while the female produces pants. Such syllables, only produced during the duet, may act as vocal markers, perhaps facilitating the synchronization between pair mates. In *P. donacophilus*, the switch from female to male dominated sections occur every ~2.5 s on average (Figure 4). The male or female pants are less audible when the mate is producing bellows and arches. Reducing the gain on the spectrogram annihilates the soft pant phrase, preserving louder syllables such as vocal markers, hoots, bellows, and arches. A duet may last five minutes or more without pause (Figure 5) but, on some occasions, it stops abruptly for a few seconds in response to a sudden disruption (e.g., an alarm call from a group member). The duet ends when one individual stops contributing while his/her partner emits a few extra syllables.

### 3.2. Organization of Duets in the Moloch Group

**Figure 6:**
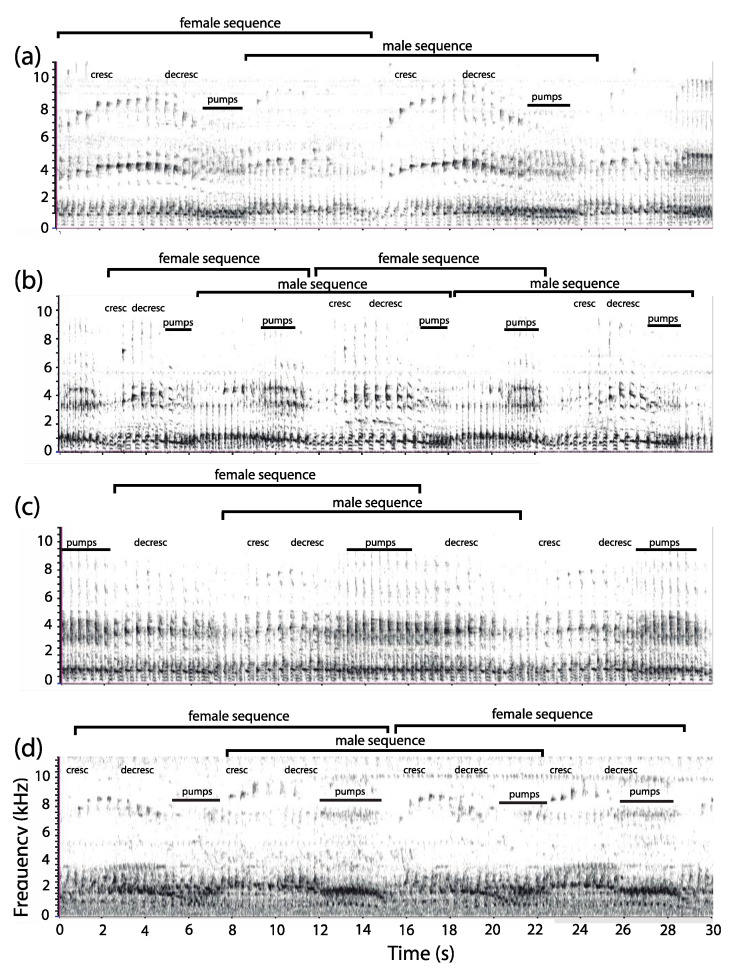
Periodic vocal duets produced in the moloch lineage. (**a**) *Plecturocebus discolor*, (**b**) *P. aureipalatii*, (**c**) *P. toppini*, and (**d**) *P. urubambensis*. Shown in each panel are overlapping male and female sequences beginning at the onset of each pant phrase. Long series of ascending and descending bellows that culminate with pumping typify the duets in the moloch lineage.

The coordinated loud calls of *P. discolor* (*n* = 8), *P. toppini* (*n* = 7), *P. aureipalatii* (*n* = 1), and *P. urubambensis* (*n* = 1)—the latter cautiously assigned to the moloch lineage—reveal a pattern similar to that previously described for *P. ornatus* [38] and *P. cupreus* [44]. A typical, periodic duet starts with a series of introductory moans, sometimes with a mix of high pitched syllables, followed by a long series of bellows produced by one pair member during which the mate contributes pant syllables (Figure 6). Both partners then reach a climax known as pumping, a short series of syncopated syllables produced in a rapid cadence (*P. aureipalatii*: 4.89 ± 0.13 calls/s, *n* = 8; *P. toppini*: 4.26 ± 0.10 calls/s, *n* = 50; *P. urubambensis*: 4.18 ± 0.04 calls/s, *n* = 4; and *P. discolor*: 4.14 ± 0.20 calls/s, *n* = 15). Without pause, the duet proceeds with a coordinated sequence of pants and bellows, combined with a sex-reversal that leads to another climax of pump syllables. As can be seen in Figure 6, the frequency of pumps seems to vary across species, with *P. aureipalatii* pumping more often than *P. discolor*. The joint emissions may continue for several minutes until the letdown, a series of honks that end up the duet. Compared with the donacophilus lineage, the moloch duets sequences have a much longer duration (>10 s), but similar call rate (*P. aureipalatii*: 3.29 ± 0.20 calls/s, *n* = 32; *P. toppini*: 2.87 ± 0.08 calls/s, *n* = 195; *P. urubambensis*: 2.81 ± 0.19 calls/s, *n* = 20; and *P. discolor*: 2.80 ± 0.07 calls/s; *n* = 146). Within a duet sequence, the male bellow phrase contains from six to twelve bellows, depending on the species (*P. discolor*: 11.97 ± 0.43, *n* = 33; *P. toppini*: 9.10 ± 0.28, *n* = 50; *P. aureipalatii*: 8.11 ± 0.48, *n* = 9; and *P. urubambensis*: 10.20 ± 0.37, *n* = 5). During the crescendo phase, the bellow call rate is slower than it is during the decrescendo phase, with a gradual change in syllable morphology. This pattern is particularly salient in *P. discolor* (mean rate: 2.15 calls/s vs. 2.53 calls/s; Wilcoxon Signed Rank test: *V* = 0; *n* = 8; *p* = 0.0078).

**Figure 7:**
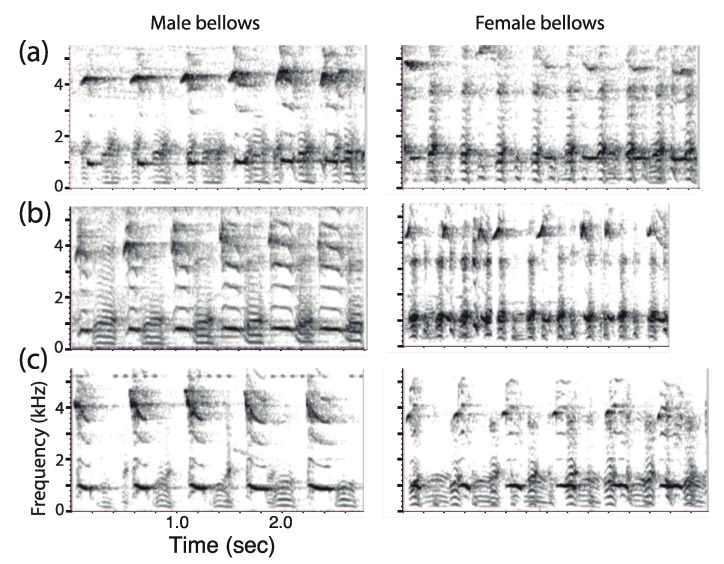
Exemplars of bellows emitted by (**a**) *P. discolor*, (**b**) *P. aureipalatii*, and (**c**) *P. toppini*. Left: putative male bellows. Right: putative female bellows. Note the joint emission of pant calls by the partner.

Analysis of the 14 power spectra produced by each adult pair revealed significant sex-related differences in the dominant frequency (Figure 7). With no exception, in each pair that we examined (*n* = 7), putative female bellow—male pant combinations exhibited a higher dominant frequency than putative male bellow—female pant combinations (*P. discolor*: 1049.7 ± 16.1 Hz vs. 931.3 ± 23.9 Hz; *n* = 4; *P. toppini*: 1044.4 ± 53.8 Hz vs. 969.0 ± 64.6 Hz; *n* = 2; and *P. aureipalatii*: 1012.1 Hz vs. 796.7 Hz; *n* = 1) (Wilcoxon Signed Rank test: *V* = 0; *p* = 0.016).

### 3.3. Organization of Duets in the Torquatus Group

**Figure 8:**
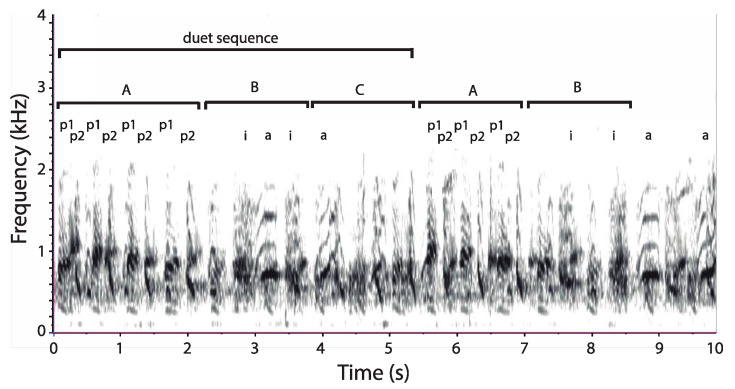
Joint vocalizations emitted by a pair of *Cheracebus lucifer*. The putative male sequence consists of three phrases (A, B, and C), which are repeated. p1 and p2 are pant syllables. i: inhaled, noisy syllables. a: putative female arch.

The coordinated loud call of *C. lucifer* (*n* = 6) has a low dominant frequency (mean: 671.1 ± 0.04 Hz). The putative male sequence consists of three distinct phrases, each composed of consecutive paired syllables. Phrase A is a short series of pant syllables (call rate: 1.94 ± 0.40 calls/s; *n* = 84). Phrase B consists of two to four inhaled, noisy syllables (call rate: 1.39 ± 0.01 calls/s; *n* = 44). The sequence ends with phrase C (call rate: 1.91 ± 0.20 calls/s; *n* = 14). The putative female sequence appears to contain two phrases, one consisting of arches (Figure 8).

### 3.4. Organization of Duets in the San Martin Titi Monkey

**Figure 9:**
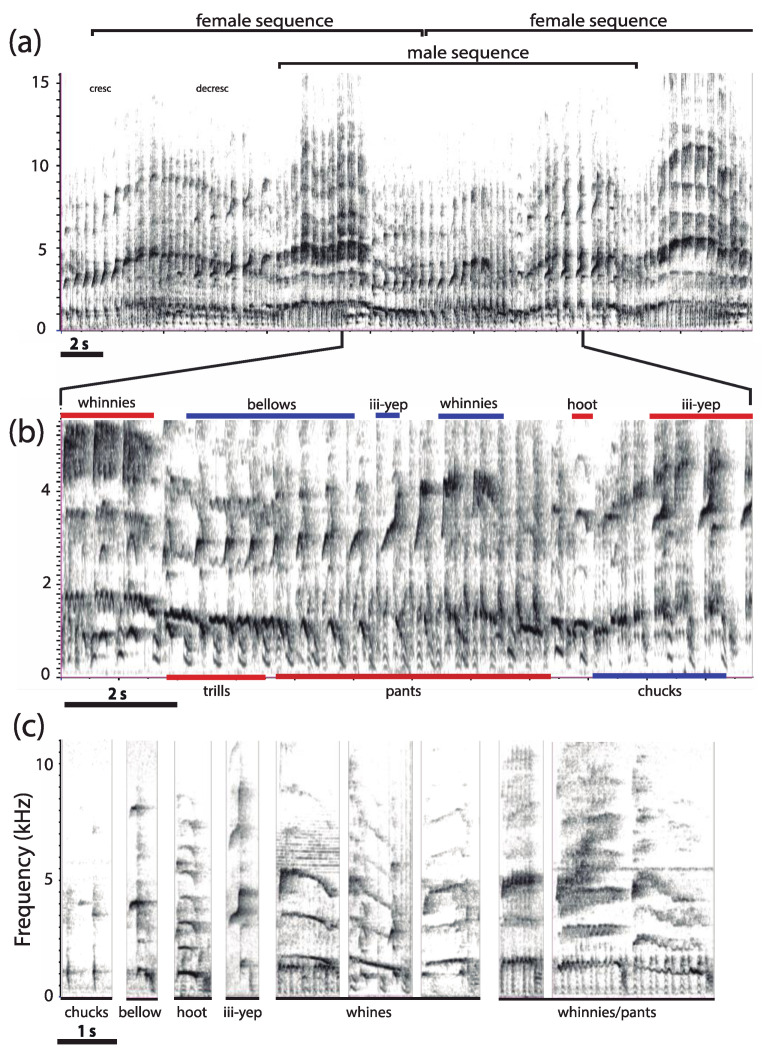
Vocal duet emitted by a pair of the San Martin titi monkey, *P. oenanthe*. (**a**) Periodic pattern showing two consecutive female sequences with overlapping male sequence. Note the crescendos of male bellows and female iii-yep syllables. The whole vocal display is accompanied by an exuberant amount of sound energy in the upper frequency range. (**b**) Segment of the same duet showing the respective contribution of the female (in red) and the male (in blue). The female first produces a series of whines, then a pant phrase followed by a series of iii-yep syllables. At the same time, the male contributes a long series of bellows that gradually merge into whines. (**c**) Examples of broadband and tonal syllables that appear to be unique to the San Martin titi monkey (iii-yep, whines, and whinnies). Many of these spectrograms contain vocalizations emitted by the two partners simultaneously.

The coordinated loud call of *P. oenanthe* (*n* = 1) reveals a high dominant frequency (mean: 1464.2 Hz) and duet sequences of long duration (~20 s). A putative female sequence consists of five phrases produced in the order: iii-yep–whinnies–trills or chucks–pants–hoots, with a characteristic crescendo and decrescendo occurring during production of female whinnies (Figure 9a). A putative male sequence consists of bellows–iii-yep–whinnies–chucks–pants–hoots. Male bellows gradually merge into a series of whinnies produced as a decrescendo, and both phrases overlap with female pants (Figure 9b). Doublets and triplets of male chucks produced in rapid cadence overlap with female iii-yep syllables (Figure 9b). Female whinnies merge into trills that overlap with male bellows (Figure 9b) or they may occur jointly with male pants (Figure 9c). Whines (Figure 9c) are produced sporadically, usually after a series of female whinnies.

### 3.5. Power Spectral Density 

**Figure 10:**
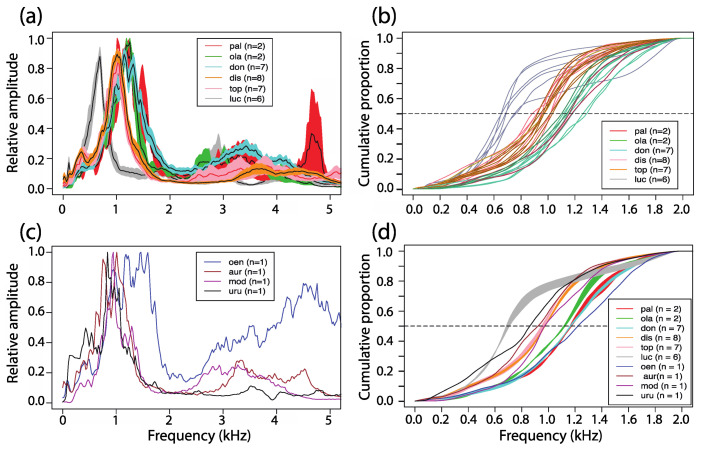
(**a**) Power spectra of vocal duets showing the distribution of sound energy as a function of frequency. The relative amplitude is shown as a proportion of the normalized signal amplitude. For each graph, the mean value (±SEMs) has been plotted and the largest peak represents the dominant frequency. The first three letters of each species’ name have been used as an acronym. The number of distinct groups that were analyzed is indicated by the n value. (**b**) The cumulative proportion of sound energy within the frequency band (80–2000 Hz) where the main peaks occurred is depicted for each duet. For statistical comparisons, frequency measurements were taken halfway up the cumulative curves (dashed line). (**c**) Power spectra of vocal duets in four species of titis for which only one group was available. Note the large rebound of sound energy at 4 to 5 kHz in the loud call of *P. oenanthe*. (**d**) For six of the ten species, the cumulative frequency plots have been averaged and superimposed together with four individual spectra revealing three clusters of species, halfway up in the frequency band of 0.6 to 1.2 kHz.

The mean power spectra of 32 duet exemplars in six species of titis for which more than one group was available differed among taxa (Figure 10a). Overall, sound energy was concentrated within the 0.5 to 1.5 kHz frequency band, with *C. lucifer* on the low side of the spectrum and *P. olallae* on the high side. Between these two extremes, the mean power spectra of *P. pallescens* and *P. donacophilus* overlapped almost completely as did the mean power spectra of *P. discolor* and *P. toppini*. Measurements of the frequency distributions halfway up the cumulative power spectra within the 80 to 2000 Hz frequency band (Figure 10b) confirmed absence of significant differences between *P. discolor* and *P. toppini* (Wilcoxon Rank Sum test: W_dis vs top_ = 20; *n*1 = 8, *n*2 = 7; *p* = 0.38). By contrast, after pooling the data from *P. discolor* and *P. toppini* in a moloch cluster, we found significant differences between *C. lucifer* and the moloch cluster (Wilcoxon Rank Sum test: W_luc vs mol_ = 0; *n*1 = 6, *n*2 = 15; *p* < 0.001) and also between *P. donacophilus* and the moloch cluster (Wilcoxon Rank Sum test: W_mol vs don_ = 0; *n*1 = 7, *n*2 = 15; *p* < 0.001). The spectrum of *C. lucifer* departed from all other spectra with 75% of the sound energy concentrated below 969 Hz.

For comparison, Figure 10c shows the power spectra of duets recorded from four additional taxa for which we had samples from one family group. The power spectrum of *P. modestus* largely overlaps with *P. aureipalatii* and *P. urubambensis* spectra in the moloch lineage. Together, these three taxa exhibit a peak of sound energy below 1 kHz. By contrast, the power spectrum of *P. oenanthe* shows three peaks shifted to the right, above 1 kHz, with an exuberant rebound of sound energy in the upper frequency range (4–6 kHz).

Superimposing all individual and averaged cumulative frequency plots obtained from ten taxa produced three main clusters: (1) a torquatus cluster with *C. lucifer*, (2) a donacophilus cluster composed of four taxa (*P. donacophilus*, *P. pallescens*, *P. oenanthe*, and *P. olallae*, the latter slightly offset), and (3) an heterogeneous cluster composed of five taxa (*P. discolor*, *P. toppini*, *P. aureipalatii*, *P. modestus*, and *P. urubambensis*) from both the moloch and donacophilus lineages. The mean frequency values obtained for each cluster halfway up the cumulative spectra were 709.90 Hz, 1177.79 ± 25.03 Hz, and 953.45 ± 19.41 Hz, respectively (Figure 10d).

### 3.6. Acoustic Parameters

#### 3.6.1. Dominant Frequency

**Figure 11:**
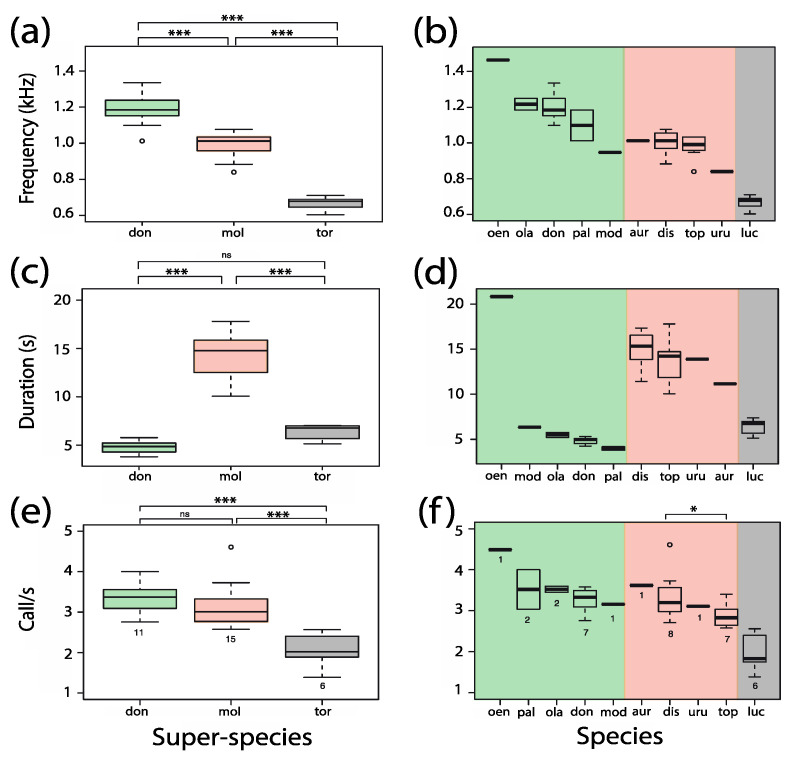
Variation in three acoustic parameters of the titis vocal duets is shown for the three lineages (left column) and the ten taxa (right column). (**a**,**b**) Dominant frequency of the joint signal, (**c**,**d**) duet sequence duration, (**e**,**f**) pant call rate. Each box displays the 1st and 3rd interquartile (25% and 75%) with the median shown by a thick horizontal bar. The whiskers denote the range and open circles are outliers. The number of duets sampled from different groups within each lineage and species is shown below the whiskers (**e–f**). Statistical analysis was performed on 32 out of 36 groups for which more than one group was available per species. The colored boxes refer to the three lineages (donacophilus, moloch, and torquatus). The first three letters of each lineage and each species are used as an acronym. Asterisks above each plot indicate significance level and ns signifies no significant difference.

The dominant frequency of 36 power spectra distributed in ten taxa ranged in a linear fashion from 603 Hz (*C. lucifer*) to 1464 Hz (*P. oenanthe*). Statistical analysis was performed on 32 power spectra obtained from six taxa for which more than one family group was available (Figure 11a). A one-way ANOVA revealed significant differences between the three lineages (F_(2,29)_ = 105.5; *p* < 0.001). The donacophilus lineage exhibited a significantly higher dominant frequency (1186.3 ± 26.2 Hz; *n* = 11) compared with the moloch (992.0 ± 17.00 Hz; *n* = 15) and torquatus lineages (667.5 ± 15.73 Hz; *n* = 6). Post-hoc comparisons with Bonferroni corrections were all significant (Student’s *t*-test: *t*_don vs. mol_ = 6.49, df = 24; *t*_don vs. tor_ = 13.7, df = 15; *t*_mol vs. tor_ = 11.2, df = 19; *p* < 0.001).

In the donacophilus lineage (Figure 11b), the duet of *P. oenanthe* had the highest median dominant frequency (1464.3 Hz), followed by *P. olallae* (1216.6 Hz), *P. donacophilus* (1184.3 Hz), *P. pallescens* (1098.2 Hz), and *P. modestus* (947.5 Hz). No significant difference was found between the three taxa for which more than one group was available (*P. olallae*: 1216.6 ± 32.3 Hz, *n* = 2; *P. donacophilus*: 1202.8 ± 30.61 Hz, *n* = 7; and *P. pallescens*: 1098.2 ± 86.13 Hz; *n* = 2) (Kruskal–Wallis Rank Sum test: χ^2^ = 1.63; df = 2; *p* = 0.44). In the moloch lineage (Figure 11b), the duets of both *P. aureipalatii* and *P. discolor* had the highest median dominant frequency (1012.1 Hz), followed by *P. toppini* (990.5 Hz) and *P. urubambensis* (839.8 Hz). There was no significant difference between *P. discolor* (1003.99 ± 22.63 Hz, *n* = 8) and *P. toppini* (978.22 ± 26.52 Hz, *n* = 7) (Wilcoxon Rank Sum test: W_dis vs top_ = 35; *p* = 0.45).

#### 3.6.2. Sequence Duration

The duration of 432 duet sequences measured across the ten taxa ranged from 3.78 s (*P. pallescens*) to 20.82 s (*P. oenanthe*). A linear mixed effects model was performed on the six taxa for which we had more than one group, yielding a total of 384 duet sequences measured from 32 groups. For each taxon, the data fitted a Gaussian distribution. The variance (2.44) and standard deviation (1.56) explained by the random effect departed from zero, indicating that the random effect was essential in the model. Relative to the intercept, *P. discolor*, the *t*-values for the fixed effect suggest there were substantial differences in duet sequence duration between taxa (*t*_dis vs. don_ = −12.03, *t*_dis vs. ola_ = −7.47, *t*_dis_ _vs. pal_ = −8.71, *t*_dis vs. luc_ = −9.65, *t*_dis vs. top_ = −1.64). In fact, analysis of deviance was significant (Wald test: χ^2^ = 249.02, df = 5, *p* < 0.001), increasing our confidence that duet sequence duration depended on the fixed effect (species). 

As shown in Figure 11c, there were significant differences between the three lineages (F_(2,29)_ = 117.3; *p* < 0.001). Post-hoc tests found significantly longer duet sequences in the moloch group (14.35 ± 0.59 s; *n* = 15) compared with the torquatus (6.39 ± 0.32 s; *n* = 6) and the donacophilus (4.78 ± 0.18 s; *n* = 11) lineages (Welch Two Sample *t*-test with Bonferroni corrections: *t*_mol vs. don_ = 15.4; *t*_mol vs. tor_ = 11.7; *p* < 0.001). No significant difference was found between the donacophilus and the torquatus lineages (*p* > 0.05). In the donacophilus lineage, the duet sequence of *P. oenanthe* exhibited the highest median duration (20.85 s), followed by *P. modestus* (6.34 s), *P. olallae* (5.49 s), *P. donacophilus* (4.97 s), and *P. pallescens* (3.98 s; Figure 11d). No significant difference was found between the three taxa for which more than one group was available (*P. olallae*: 5.49 ± 0.28 s, *n* = 2; *P. donacophilus*: 4.83 ± 0.16 s, *n* = 7; and *P. pallescens*: 3.98 ± 0.19 s; *n* = 2) (Kruskal–Wallis Rank Sum test: χ^2^ = 5.96; df = 2; *p* = 0.051). In the moloch lineage, *P. discolor* exhibited the highest median duration (15.35 s), followed by *P. toppini* (14.23 s), *P. urubambensis* (13.89 s), and *P. aureipalatii* (11.16 s; Figure 11d). In the two taxa for which more than one group was available, the duration of duet sequences did not differ significantly between *P. discolor* (15.04 ± 0.70 s) and *P. toppini* (13.61 ± 0.97 s) (Wilcoxon Rank Sum test: W_dis vs top_ = 40; *p* = 0.19).

#### 3.6.3. Pant Call Rate

Call rate measurements from 309 pant phrases distributed across the ten taxa ranged from 1.24 call/s (*C. lucifer*) to 5.04 call/s (*P. oenanthe*) in a nonlinear fashion. A linear mixed effects model analysis was performed on six taxa, yielding a total of 265 call rate measurements from 32 groups. The variance (0.17) and standard deviation (0.41) explained by the random effect differed from zero, again suggesting it was essential in the model. Relative to the intercept, *P. discolor*, the *t*-values for the fixed effect suggest there were substantial differences in pant call rate between taxa (*t*_dis vs. don_ = −0.29, *t*_dis vs. ola_ = 0.60, *t*_dis vs. pal_ = 0.60, *t*_dis vs. luc_ = −5.96, *t*_dis vs. top_ = −2.02). Analysis of deviance was significant (Wald test: χ^2^ = 51.11, df = 5, *p* < 0.001), increasing our confidence that pant call rate depended on the fixed effect.

As shown in Figure 11e, one-way ANOVA on the log-transformed data (Shapiro–Wilk: *p* > 0.1; Levene test: F_(2,29)_ = 0.99; *p* > 0.05) revealed significant differences between the three lineages (F_(2,29)_ = 23.5; *p* < 0.001). Compared with the torquatus lineage (2.05 ± 0.17 calls/s; *n* = 6), pant call rate was significantly higher both in the donacophilus and the moloch lineages (3.35 ± 0.10 calls/s; *n* = 11 and 3.13 ± 0.14 calls/s; *n* = 15, respectively) (pairwise *t*-test with Bonferroni corrections: *t*_don vs. tor_ = 6.6, df = 15; *t*_mol vs. tor_ = 5.2, df = 19; *p* < 0.001). There was no significant difference between the donacophilus and the moloch lineage (*t*_don vs. mol_ = 1.4, df = 24; *p* > 0.05). In the donacophilus lineage (Figure 11f), the duet of *P. oenanthe* had the highest median call rate (4.49 calls/s) followed by *P. olallae* (3.53 calls/s), *P. pallescens* (3.52 calls/s), *P. donacophilus* (3.33 calls/s), and *P. modestus* (3.16 calls/s). No significant difference was found between the three taxa for which more than one group was available (*P. pallescens*: 3.52 ± 0.48 calls/s, *n* = 2; *P. olallae*: 3.53 ± 0.07 calls/s, *n* = 2; and *P. donacophilus*: 3.33 ± 0.11 calls/s; *n* = 7) (Kruskal–Wallis Rank Sum test: χ^2^ = 1.65; df = 2; *p* = 0.44). In the moloch lineage (Figure 11f), the duet of *P. aureipalatii* had the highest median call rate (3.62 calls/s) followed by *P. discolor* (3.20 call/s), *P. urubambensis* (3.11 call/s), and *P. toppini* (2.83 call/s). Pant call rate was significantly higher in *P. discolor* (3.35 ± 0.21 calls/s, *n* = 8) as compared with *P. toppini* (2.88 ± 0.11 calls/s, *n* = 7) (Wilcoxon Rank Sum test: W_dis vs top_ = 46; *p* < 0.05).

### 3.7. Discriminant Function Analysis 

**Figure 12:**
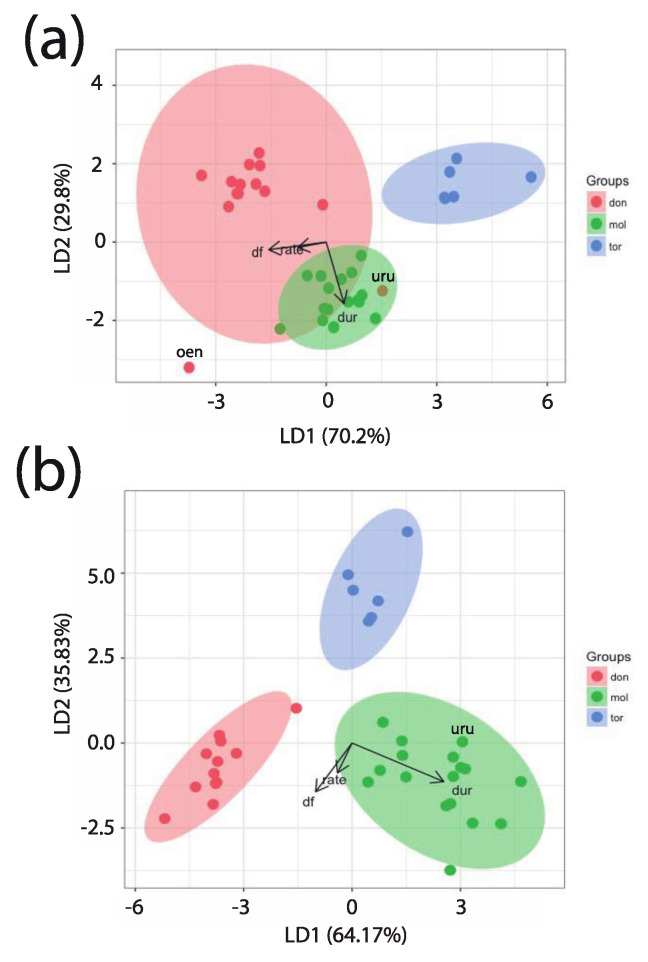
Ordination biplots obtained from the linear discriminant analysis (LDA) output. (**a**) Analysis performed with *P. urubambensis* and *P. oenanthe* both assigned to the donacophilus lineage. The two misclassified data points have been labeled by their acronym. (**b**) Analysis performed with *P. urubambensis* assigned to the moloch group (uru), and *P. oenanthe* being excluded. The proportion of separation achieved by each discriminant function (LD1 and LD2) is shown in brackets. Each data point represents a score resulting from the linear combination of the three predictors that best separates the three lineages. The scatter around each centroid is such that there is considerable overlap (top) or no overlap (bottom) between lineages. The shape of the 95% confidence ellipse around each centroid depicts the influence of each of the three predictors on the within-group variance. The loadings of each predictor variable are plotted as arrows (or vectors) labeled with the name of the predictor variable. Arrow length indicates the variance and the angle between arrows portrays the degree of correlation between predictors. For instance, the small angle between df and rate reflects a high correlation between these two variables, with df and dur exhibiting larger variances compared with rate. Both df and rate are almost at right angle with dur, which signifies the absence of correlation between these pairs of variables. The two dots just outside the 95% confidence ellipses in panel b are from *P. modestus* (red dot) and *P. discolor* (green dot).

With *P. oenanthe* and *P. urubambensis* assigned to the donacophilus lineage, the within-group normality assumption was not met for one of the three predictors, even after data transformation (Shapiro–Wilk: W = 0.7142, *p* < 0.001). Pant call rate was correlated with dominant frequency (Pearson’s product-moment correlation: *t* = 5.85, *r* = 0.71; df = 34, *p* < 0.001). Boxplots of standardized values within each group revealed the presence of five outliers. Although the first two linear functions accounted for the total variation in the dataset (LD1: 70.2% and LD2: 29.8%), there was considerable overlap between the donacophilus and moloch lineages (Figure 12a). We also found two misclassifications in the confusion matrix (*P. oenanthe* and *P. urubambensis*).

With *P. oenanthe* removed from the analysis and *P. urubambensis* assigned to the moloch lineage, and after data transformation, the within-group normality assumption was met for each predictor on the standardized values (Shapiro–Wilk: W = 0.903, *p* > 0.07). Pant call rate was still correlated with dominant frequency (Pearson’s Product-Moment Correlation: *t* = 4.36, *r* = 0.60; df = 33, *p* < 0.001). We performed the LDA despite the presence of one outlier—a *P. discolor* subject panting at an elevated rate. While LD1 (64.17%) resulted in a partial dissociation of the groupings, together with LD2 (35.83%) maximal separation (100%) was achieved between the three lineages (Figure 12b). In fact, we found no misclassification in the confusion matrix. Subsequent MANOVA performed on this dataset revealed that our discriminant analysis was highly significant (Wilks Lambda = 0.017; F_(2,32)_ = 66.3; *p* < 0.001). Post-hoc comparisons between pairs of lineages were all significant (Pillai_don vs. mol_ = 0.90, F_(1,27)_ = 73.2; Pillai_don vs tor_ = 0.93, F_(1,16)_ = 58.9; Pillai_mol vs. tor_ = 0.88, F_(1,21)_ = 46.3; *p* < 0.001). In other words, using only three acoustic parameters, LDA correctly allocated loud calls of titi monkeys to their respective group membership.

## 4. Discussion

### 4.1. Form and Structure of Titi Monkey Duets

The study aimed at exploring the diversity of callicebine loud calls among ten species of titis distributed in three of the four main clades currently recognized [62]. Four basic patterns of duetting—donacophilus, moloch, torquatus, and oenanthe patterns—emerged from our acoustic analysis (Supplementary Material). First, the donacophilus pattern is characterized by a relatively high dominant frequency, short duet sequences consisting of high call rates, five or six coordinated phrases with a female-dominated section preceding a male-dominated section. Second, the moloch pattern exhibits a lower dominant frequency and features duet sequences of much longer duration but with call rates similar to the donacophilus pattern. The long series of bellows produced as a crescendo and decrescendo that culminate with pumping represent a striking feature of the duetting pattern in the moloch lineage [37,38]. Pumping does not occur in the donacophilus lineage, but has been reported in the torquatus lineage [83]. Third, the vocal duets of *Cheracebus lucifer*, the only representative taxon of the torquatus lineage in our study, stand out from the other two lineages, both in the spectral and temporal domains. The duet sequence of *C. lucifer* is composed of a reduced number of song phrases having a low syllable rate. With a body mass of 1100 to 1500 g, *C. lucifer* is the largest of all callicebine monkeys, which might explain the lower pitch of its voice. Kinzey and colleagues [83] provide a detailed account of the species’ vocalizations, but their study is devoid of spectrograms. Based on the available material, we could not ascribe the calls produced by this titi to those described in the donacophilus and moloch lineages, except for pant syllables. Further studies are needed to better characterize the duetting patterns among the six *Cheracebus* members of the torquatus lineage. Fourth, *P. oenanthe* produces the most distinctive and elaborate duet of all callicebine monkeys studied to date. In the temporal domain, it features the longest sequence duration and the highest pant call rate among the ten taxa that were examined. In the frequency domain, in addition to the production of broadband calls typical of other titi monkeys (e.g., pants and bellows), a large subset of the *P. oenanthe* vocal repertoire consists of narrowband sounds, such as whinnies, whines, and iii-yep syllables that appear to be unique to this species [80]. Consistent with this phonological distinction, the duet power spectrum displays the highest dominant frequency and shows a substantial rebound of sound energy around 4 to 6 kHz, unseen in other taxa. Overall, the duetting pattern of *P. oenanthe* appears to combine acoustic features reminiscent of both the moloch and donacophilus groups. The crescendo of whinnies, produced at a slow rate, followed by a decrescendo of similar syllables produced at a faster rate, is typical of *P. discolor* in the moloch group. Unlike the latter, however, the decrescendo does not lead to pumping, which, apparently, is not part of the species’ repertoire. On the other hand, panting sounds very similar to other members of the donacophilus group, with the exception of *P. urubambensis*. 

Compared with the continental distribution of the callicebine monkeys, the study was conducted over a relatively small geographic scale (~10%) so that additional patterns of duetting may be found in other regions of the Amazon Basin and in eastern Brazil. In particular, with the exception of *C. nigrifrons* [48], little is known on the duetting patterns in the personatus lineage for which recent molecular studies have shed the light on the time of divergence, and the phylogenetic and biogeographic relationships among all five *Callicebus* species [66].

As reported by Robinson [38], we were able to detect subtle but reliable sex-related differences in the calls of *P. discolor*, *P. toppini*, and *P. aureipalatii* within the moloch lineage. In particular, sex determination during production of the pant-bellow phrase combination by the two pair members was crucial to understand the temporal organization of the duet. Specifically, putative male bellows were characterized by lower dominant frequency than putative female bellows. From his study with wild *P. ornatus*, Robinson [38] reported that “Female pumps have a higher dominant frequency at the mid-point of the climax than those of males” and “the softer female bellows have a distinguishable difference in tonal quality, a slightly lower dominant frequency at mid-duration…and are shorter” (p. 390). Working with captive *P. cupreus*, Müller and Anzenberger [44] were unable to confirm Robinson’s findings. At this point, we do not have a clear explanation as to why the sex-related difference in the dominant frequency of bellows goes in the opposite direction for *P. ornatus* [38]. In spite of our lack of knowledge on the auditory capacities of titi monkeys, it would not be surprising that these primates are capable of sex recognition based on auditory cues alone.

Although we focused on the acoustic component of this collective behavior [32], duetting is a multimodal display involving complex sequences of nonvocal signals, such as facial expressions, body movements (e.g., arching the back, bowing of the arms, body shaking, tail lashing, and piloerection [52]), and extensive locomotor activity (jumping over or moving toward or away from a partner), depending on the context, and the species. For instance, in the moloch lineage (*P. cupreus* and *P. ornatus*), it has been reported that pair mates stay close to one another and cannot duet if separated by more than one meter [38,44,52]. By contrast, in the donacophilus lineage (*P. donacophilus*, *P. olallae*, and *P. modestus*), we observed, on many occasions, the two pair mates duetting at different heights or running and leaping through the trees while calling jointly several meters from one another during intergroup encounters. Such behavior does occur in *P. discolor* and *P. aureipalatii*, although very rarely (S.M.V.K. and J.M.M., pers. com.). The male dawn call of *C. lucifer* is accompanied by long runs and leaps through the trees, with the female following the male passively [83]. In the personatus lineage, when group members of *C. nigrifrons* are dispersed, the short and cyclic call (type 1) is produced mostly as a chorus before the group reunites; the long and noncyclic call (type 2) is emitted as a duet, with both pair mates maintaining tight physical contact (C.B.C., pers. com. [48]). These anecdotal observations might provide an incentive for comparative studies on the function of duetting among callicebine monkeys that is not just limited to the acoustic channel. The side-by-side vocal display may sharpen the timing of the duet coordination, based on auditory feedback [28], possibly conveying information to listeners on the strength of the pair bond and the pair commitment to defend the territory [29].

We are not aware, either in birds or mammals, of bioacoustics reports in which the power spectral analysis of a combined signal—here considered as a functional entity—has been exploited for the profit of comparative studies. Such analysis is usually performed on the vocalizations emitted by a single individual [104], resulting in an accurate distribution of sound energy as a function of frequency. Accordingly, some of our density power spectra showed two, slightly off, peaks of sound energy on the frequency axis, which might correspond to the respective contribution of each pair member to the duet. Overall, this procedure turned out to be productive in revealing three clusters of cumulative frequency spectra generated from our 36 duet samples distributed across ten callicebine taxa. However, the respective composition of each cluster did not squarely match each of the three recognized lineages, pointing to some inconsistencies in our dataset.

This was confirmed in subsequent linear discriminant analyses, which revealed the presence of two outliers within the donacophilus lineage. Eliminating one of these outliers (*P. oenanthe*) from the analysis and re-allocating the other (*P. urubambensis*) to the moloch lineage resulted in clear separation of the taxonomic groupings. Of the three predictors, duet sequence duration weighted more in separating the moloch from the donacophilus–torquatus lineages, whereas both the dominant frequency of the combined signal and pant call rate weighted more in separating the torquatus from the donacophilus–moloch lineages. The unique duetting pattern of *P. oenanthe* precluded its inclusion in the LDA and thus represents a fourth category in its own right.

### 4.2. Titi Monkey Duets and the Taxonomy of the Callicebinae

The form and structure of titi monkey loud calls examined in the present study raise important questions with respect to the current callicebine taxonomy [62,63,64,65,66]. First, within the donacophilus group, *sensu largo*, we found the pattern of duetting of the two Peruvian endemics *P. oenanthe* and *P. urubambensis* to differ substantially from the other four members of the donacophilus lineage. The recently discovered Urubamba brown titi [72] was added to the donacophilus lineage based on its body size and coat color (J.V., pers. com.). Here, however, we have shown that the loud calls of this species share affinities with vocal patterns in the moloch group, calling into question the tentative placement of *P. urubambensis* in the donacophilus lineage. Until further genetic studies are conducted, it would seem logical to consider the donacophilus lineage *sensu stricto* as a grouping of four *Plecturocebus* species (*donacophilus*, *pallescens*, *olallae*, and *modestus*), i.e., excluding both *P. urubambensis* and *P. oenanthe*.

The power spectrum of *P. modestus* vocal duet indicates that even this taxon departs from closely related species within the donacophilus group, including *P. olallae*, *P. donacophilus*, and *P. pallescens*. *P. modestus* loud calls are characterized by a relatively slow call rate, longer duet sequence and a dominant frequency below 1 kHz, similar to that of *P. urubambensis*. This view was reinforced in our linear discriminant analysis in which *P. modestus* appears to be most distant from the group centroid. Indeed, in his extensive taxonomic revision of the previous *Callicebus* genus, Hershkowitz [56] wrote: “*Callicebus modestus*… stands apart from all congenerics by its elongate skull and extremely small braincase”. Given the evidence, the genetic makeup of *P. modestus* may prove to differ from other *Plecturocebus* (*olallae*, *donacophilus*, and *pallescens*) in the donacophilus lineage, *sensu stricto*. To date, however, estimates of the fixation index F_ST_ derived from scats and blood samples found less genetic differentiation between *P. modestus* and *P. olallae* than between the latter two and *P. donacophilus* [105].

Second, the pattern of duetting in the donacophilus lineage, *sensu stricto*, sharply contrasts with that described for the moloch lineage, including *P. ornatus* [38], *P. cupreus* [37,44], and the four additional *Plecturocebus* (*discolor*, *toppini*, *aureipalatii*, and *urubambensis*) reported in this study. Ten of the 16 recordings of the Macaulay library that form part of our dataset belong to the moloch lineage. Of those, seven were originally reported as being of *Callicebus moloch*, subsequently as *C. brunneus*, and now considered as *P. toppini*. The five recording locations of *C. moloch* at Tambopata research center (n = 3), Manu National Park (n = 1), and Los Amigos field station (n = 1) are part of a whole area in southern Peru occupied by *P. toppini*, which, only recently, was reinstated as a full species [72]. The three recordings of titis from Ecuador, originally reported as being of *Callicebus cupreus*, are now identified as *P. discolor* of the moloch lineage, according to the current callicebine taxonomy [62]. Notably, *P. discolor* duetting pattern closely matches that of *P. toppini* despite the fact that both species were formerly classified in two separate lineages (cupreus and moloch, respectively) [59]. Furthermore, the loud call of *P. aureipalatii*, a species discovered by Wallace and colleagues [68], shares an overall similarity with both *P. discolor* and *P. toppini*. *P. aureipalatii*, however, differs from both by exhibiting shorter duet sequences and higher pant call rates. Hoyos and colleagues [64] did not include *P. aureipalatii* in their genetic analysis “due to a lack of formal placement within any one species group”.

Despite a short recording, we cautiously assigned *P. urubambensis* to the moloch group because of a strong similarity of the species’ duet with this lineage. Our goal is not to propose a new taxonomy but to emphasize the need for molecular studies in this taxon. It will be very interesting if *P. urubambensis* was indeed genetically related to the donacophilus group, and yet produces a moloch-like duetting pattern, suggesting a socially mediated influence or an adaptation to the physical environment. As an alternative, if genetic data were to indicate that *P. urubambensis* belongs to the moloch group, it would provide satisfying confirmation of the notion that loud calls are useful behavioral markers of phylogeny [77,78,106]. The duet recording of *P. urubambensis* was obtained from one pair in central Peru, where the species occurs alone whereas in Southern Peru, *P. urubambensis* is sympatric with *P. toppini* [72]. The concept of character displacement predicts that the phenotypes of two closely related species with an overlapping geographic range will diverge where both species are sympatric and will converge where each species occurs alone [107]. Comparing the duetting patterns of these two titis in both sympatric and allopatric areas could be revealing.

Third, in the torquatus lineage, the duet of *C. lucifer* is characterized by a low dominant frequency, fewer phrases and the emission of noisy syllables produced at a slow rate. These features, together, may represent primitive acoustic traits. Recent genetic analysis using both mitochondrial and nuclear gene markers found *C. lucifer* and *C. purinus* more closely related to each other than they are to *C. lugens* [65]. Together with *C. torquatus*, *C. regulus*, and *C. medemi*, these six taxa form a basal grouping—the genus *Cheracebus*—that diverged early on from the common ancestor that gave rise to the wide callicebine radiation as we know it today [62].

Fourth, *P. oenanthe* was originally classified in the donacophilus group because of shared anatomical and morphological attributes with *P. donacophilus* [56,57,59,72]. Referring to the medium class II of titi monkeys, which is based on craniocerebral criteria, Hershkowitz noted ”All members of this class are closely related inter se, but with a greater distance between *oenanthe* and the rest” [57]. In that respect, *P. oenanthe* shares similarities with *P. modestus* (see above). Nevertheless, the duetting pattern of *P. oenanthe* is so divergent from *P. modestus* homologous loud call that we consider this taxon distinct from the donacophilus group, *sensu stricto*.

To sum up, our findings generally agree with Kobayashi’s classification scheme [58] in which the donacophilus lineage comprises the four *Plecturocebus* taxa *olallae*, *modestus*, *donacophilus*, and *pallescens*, with the latter two being considered as subspecies. Unlike Kobayashi’s classification, however, we consider *donacophilus* and *pallescens* as distinct species. The acoustic evidence presented here also supports Hershkowitz’s [57] conclusion that, within this clade, *P. modestus* singles itself out, suggesting that genetic studies of the position of *P. modestus* are needed [105] and that this taxon may differ from other members of the donacophilus lineage, *sensu stricto* [108]. Only three out of the ten taxa (30%) examined in this study have been included in phylogenetic studies using genetic data [62,64,65]. The recent study by Byrne and colleagues [62] on the former genus *Callicebus* was performed with nuclear and mitochondrial DNA material obtained from *C. donacophilus*. Further molecular studies using high quality DNA samples from other taxa in the donacophilus lineage will be necessary to confirm or reject our conclusions.

### 4.3. Titi Monkey Duets and Environmental Influences

In addition to genetic factors, selective pressures from the physical and/or social environment may also determine the form and structure of vocalizations [11,109]. Our study was conducted in a variety of habitats ranging from dry riparian forests of the Chaco, to gallery forests of the Bolivian pampas, to remote Amazon rainforest in Peru and Ecuador. However, studies of sound transmission in those habitats are still lacking, which could have helped us relate the titis’ loud call structure to acoustic properties of their respective habitat. As a special case, the Peruvian endemic *P. oenanthe* exploits a variety of altitudinal habitats, ranging from humid to dry forests and can be found as high as 1300 m [72]. Our recording from *P. oenanthe* northern population was made at an altitude of 850 m. The mountains of the San Martin Department provide a physical barrier for this critically endangered titi monkey. Given its restricted distribution range, we may expect very little gene flow between the northern and southern populations, a situation exacerbated by heavy deforestation [110,111,112]. Over time, genetic drift might lead to vocal divergence between these two isolated populations of *P. oenanthe*. Although it is known that *P. oenanthe* and *P. discolor* can hybridize in captivity (J.V., pers. com.), there is no evidence of hybridization in the San Martin Department of northeastern Peru where both species are sympatric [110]. The singular *P. oenanthe* pattern of duetting exhibits a large proportion of narrow-band, whistled signals that may have resulted from selective pressures impinging on these space-restricted populations in northern Peru, and may represent an adaptation for long-range communication. Indeed, some human cultures living on steep terrain are known for their elaborate whistled language as a means to communicate across valleys. “El Chiflido” in the Oaxaca Mountains of Mexico, “El Silbo Gomero” in the Canary Islands, or the recently extinguished “Aas whistled language” in the French Pyrenees are just a few examples of many around the world [113,114]. Future studies should investigate the acoustic properties of the habitat of *P. oenanthe*, particularly in those few remote patches of forest that are still intact.

Although the vocal repertoire of *P. oenanthe* may have been shaped in part for optimal sound transmission, we do not exclude socially mediated influences on call structure. However, with few exceptions, little attention has been paid to the role of social learning in the vocal communication of callicebine monkeys. Müller and Anzenberger [44] experimented with newly formed adult pairs of *P. cupreus*, and found that they were able to produce spontaneously a species-specific duet, although some practice was needed until they could perform a more potent duet. Robinson followed a newly formed pair of *P. ornatus,* and reported no evidence of spontaneous duetting, although both adults moaned and made some attempts at calling jointly [38]. Thus, performing a pair-specific duet in titis may require some learning skill, perhaps giving the offspring an incentive to experiment as they often join the adult pair to form a chorus (pers. obs. [48]). Far more work on social learning has been conducted on callitrichine monkeys with a cooperative breeding system, such as tamarins and marmosets. These small family-living, pair bonded neotropical primates do not duet but reliably demonstrate vocal flexibility, both in production and usage [49]. Call convergence, babbling-like behavior, and dialects are the hallmarks of vocal plasticity in these neotropical primates [49,115,116]. Geographical variations in call structure between populations of pygmy marmosets (*Cebuella pygmaea*) have been linked to social learning [49], although differences in acoustic properties across habitats partly explained such vocal variations [117]. Whether similar variations in the titis’ loud calls may be found between populations of a given species should be explored. A case in consideration is *P. discolor*, for which we recorded unusually high pant call rates from one individual in central Peru, relative to rates recorded in Amazonian Ecuador.

### 4.4. Implications for Conservation Management

Titi monkeys are shy and discrete animals exhibiting cryptic behavior. Nonhabituated family groups display highly evasive behavior in response to the presence of human observers, which makes them notoriously difficult to observe over long periods [118]. Because the habituation process of just one family group can take months, methods relying on either auditory detection of loud calls at fixed points or playback of titis’ vocalizations have been used as surrogate techniques for field surveys [94,118,119,120,121]. Better density estimations of cryptic species obtain when taking into account the seasonal variation in loud call emissions [93] and by combining playback with point transect distance methods [122]. The present study will make for a better taxonomic identification of titi monkey loud calls and for selecting the appropriate auditory stimulus for playback in field surveys of population densities. A standard call survey protocol would be welcome [87,121]. The study also underlines the taxonomic uniqueness of *P. oenanthe*, further emphasizing the importance of effective conservation actions for this critically endangered species.

## 5. Conclusions

Our comparative study of titi monkey loud calls is rooted in classical ethology [123]. As Tinbergen [124] framed it more than half a century ago, “…the trend, here, is to apply very much the same methods as those employed by taxonomists…” i.e., by “…treating behaviour patterns as “organs”… in order to establish “…classifications based on behaviour taxonomy” (p. 428).

In line with this approach, we sought for acoustic similarities and dissimilarities of titi monkey loud calls across a wide variety of habitats and species along the western edge of the Amazon and Chaco ecoregions in South America. We substantiated our descriptions of duets, based on spectrographic analysis, with power spectral analysis, quantitative measures of acoustic parameters and linear discriminant analysis. Our findings can be summarized as follows:

(1) The sharp contrast in duet organization between the donacophilus group, *sensu stricto* (*P. donacophilus*, *P. olallae*, *P. modestus*, and *P. pallescens*), the moloch group (*P. discolor*, *P. toppini*, and *P. aureipalatii*), and the torquatus group (*C. lucifer*) appears to be consistent with the current taxonomy.

(2) The six species of titi monkeys currently classified in the donacophilus group, *sensu largo*, exhibit an unexpected heterogeneity of vocal phenotypes. The occurrence of three distinct patterns of duets—donacophilus, moloch, and oenanthe—in this clade a priori suggests a taxonomic incoherence, although we do not discard a potential influence of social factors and/or habitat characteristics given the large geographical gap separating the Peruvian endemics *P. oenanthe* and *P. urubambensis* from the donacophilus group, *sensu stricto*.

(3) The duet of *P. urubambensis* conforms to the moloch lineage and the species partly shares the same habitat characteristics (lowland rainforest) with *P. toppini* in Southern Peru, which raises doubts on its taxonomic placement in the donacophilus group, based solely on physical characteristics. 

(4) The unique vocal phenotype of *P. oenanthe*, provides an interesting case to explore whether the loud call has been shaped by the acoustic characteristics of the habitat, or whether the genetic makeup of this taxon differs in unexpected ways from other closely related titis.

## Figures and Tables

**Table 1:** List of the Macaulay Library Catalogue recordings analyzed in the present study. *P*: *Plecturocebus*; *C*: *Cheracebus*. + duet followed by a distant chorus; ++ duet, not a chorus. A hyphen signifies “no information was available”.

ID	Species	Location	Country	Context	Recordist	Genus	Species *
24308	*moloch*	Madre de Dios	Peru	advertising call	T.A. Parker, III	*P*.	*toppini*
30000	*moloch*	Madre de Dios	Peru	response to playback	T.A. Parker, III	*P*.	*toppini*
30709	*moloch*	Loreto	Peru	advertising call	T.A. Parker, III	*C*.	*lucifer*
39016	*moloch*	Madre de Dios	Peru	advertising call	T.A. Parker, III	*P*.	*toppini*
39551	*moloch*	Madre de Dios	Peru	-	M.L. Isler	*P*.	*toppini*
53419	*cupreus*	Napo	Ecuador	-	L.F. Kibler	*P*.	*discolor*
75934	*moloch*	Madre de Dios	Peru	advertising call	C.A. Marantz	*P*.	*toppini*
77771	*cupreus*	Napo	Ecuador	advertising call	M.B. Robbins	*P*.	*discolor*
126317	*moloch*	Madre de Dios	Peru	advertising call	A.B. van den Berg	*P*.	*toppini*
131447	*moloch*	Madre de Dios	Peru	advertising call	J.H. Barry	*P*.	*toppini*
148573	*cupreus*	Orellana	Ecuador	-	G.F. Seeholzer	*P*.	*discolor*
187992	*moloch*	Loreto	Peru	advertising call	P.K. Donahue	*C*.	*lucifer*
188515	*moloch*	Loreto	Peru	troop chorus+	P.K. Donahue	*C*.	*lucifer*
188546	*moloch*	Loreto	Peru	troop chorus++	P.K. Donahue	*C*.	*lucifer*
188610	*torquatus*	Loreto	Peru	-	P.K. Donahue	*C*.	*lucifer*
188624	*torquatus*	Loreto	Peru	-	P.K. Donahue	*C*.	*lucifer*

* The species’ scientific name, based on recent revisions of the callicebine taxonomy [62,64,72].

**Table 2:** A list of titi monkey calls described in the literature. The first three letters of each clade and each species are used as an acronym. The original studies on which the table was elaborated have been referenced with a superscript: ^1^-[37], ^2^-[38], ^3^-[52], ^4^-[44], ^5^-[48], ^6^-[97], ^7^-[80], ^8^-[83], ^9^-[50], ^10^-[86], and ^11^-[this study]. Capital letters in the first two columns are call categories and call combinations distinguished by Moynihan [37] and Robinson [38]. R: resonating notes; P: pumping; SS: short sequence; LS: long sequence; CP: chirrup-pumping; CR: chirrup-panting; CRP: chirrup-panting-pumping. Gobble and CP are the same calls. For each species, presence or absence of a call is reported as + or − respectively. Cells for which the corresponding call might be present but has not been reported are left blank.

Category	Call	Donacophilus						Moloch					Personatus	Torquatus
		*don* ^10,11^	*pal* ^11^	*ola* ^11^	*mod* ^11^	*oen* ^7,11^	*uru* ^11^	*dis* ^1^	*orn* ^2,3^	*cup* ^1,4^	*aur* ^11^	*top* ^9,11^	*nig* ^5,6^	*luc* ^8,11^
R	pant	+	+	+	+	+	+	+	+	+	+	+	+	+
R	honk	+	+	+	+	+	+	+	+	+	+	+	+	
R	bellow	+	+	+	+	+	+	+	+	+	+	+	+	
P	pump	−	−	−	−	−	+	+	+	+	+	+	+	+
	hoot	+	+	+	+	+								+
	arch *	+	+	+	+									+
	te *	+	+	+	+									
	rhythmic *	+	+	+	+									
	chirrup	+	+	+	+	+		+	+			+	+	+
	high-pitched	+	+	+	+			+	+	+	+			
	moan			+	+	+		+	+	+				
	warble											+		
	whistle	+				+		+	+	+		+		
	squeak	+								+			+	
	chirp	+											+	
	trill	+				+		+		+				
	cheep	+											+	
	chuck					+		+		+				+
	scream	+						+	+	+				
	grunt								+	+				
	iii-yep *	−	−	−	−	+	−	−	−	−	−	−	−	−
	whine	−	−	−	−	+	−	−	−	−	−	−	−	−
	whinny	−	−	−	−	+	−	−	−	−	−	−	−	−
	pant-hoot	−	−	−	−	+	−	−	−	−	−	−	−	−
SS	gobble	+	+	+	+	+		+	+	+	+	+	+	
SS	CP	+	+	+	+	+		+	+	+	+	+	+	+
SS	CR								+					
SS	CRP								+					
LS	hoot song													+
LS	m. calling	+	+	+	+	+	+	+	+	+	+	+	+	+
LS	f. calling								+					
LS	duet	+	+	+	+	+	+	+	+	+	+	+	+	+
LS	chorus	+	+	+	+	+	+	+	+	+	+	+	+	+

* additional titi monkey calls identified in this study.

**Table 3:** The ten species of titi monkeys from which duets and choruses were analyzed. For each species, the number of analyzed duets and choruses are reported together with the total duration of the recordings and the media that was used (v = video, a = audio). Gray areas highlight total numbers separately for the donacophilus and moloch lineages. *P*. = *Plecturocebus*; *C*. = *Cheracebus*. Species for which genetic studies exist [62,64,65] are designated by the superscript ++. Hyphen signifies “no data”.

Genus	Lineage	Species	#Groups	#Duets	#Choruses	Dur (s)	Media
*P*.	donacophilus	*donacophilus ^++^*	7	6	1	798	v
		*pallescens*	2	2	-	283	v
		*olallae*	2	2	-	230	v
		*modestus*	1	1	-	173	v
		oenanthe *	1	1	-	189	a
		uubambensis *	1	1	-	75	v
	moloch		14	13	1	1748	
		*toppini*	7	6	1	810	a
		*discolor ^++^*	8	6	2	801	a/v
		*aureipalatii*	1	1	-	127	a
			16	13	3	1738	
*C*.	torquatus	*lucifer ^++^*	6	5	1	618	a
		Total	36	31	5	4104	

* An asterisk denotes a species with a distinct vocal phenotype (this study).

## Supplementary Materials

The following are available online at https://www.mdpi.com/2076-2615/8/10/178/s1, Video S1: Duetting Patterns of Titi Monkeys: donacophilus, moloch, torquatus, oenanthe.

## Appendix

The following recordings at the Macaulay Library at the Cornell Lab of Ornithology were referenced: 24308, 30000, 30709, 39016, 39551, 53419, 75934, 77771, 126317, 131447, 148573, 187992, 188515, 188546, 188610, 188624.
